# Effect of Personal Protective Equipment (PPE) on Surgical Site Infection in Emergency Laparotomy: An Observational Study From a Tertiary Care Centre

**DOI:** 10.7759/cureus.24278

**Published:** 2022-04-19

**Authors:** Rohik Anjum, Farhanul Huda, Aakansha G Goswami, Amoli Tandon

**Affiliations:** 1 General Surgery, All India Institute of Medical Sciences, Rishikesh, IND

**Keywords:** emergency laparotomy, emergency surgery, ppe, personal protective equipment, surgical wound infection, postoperative wound infection, surgical site infection

## Abstract

Background

In the era of the coronavirus disease 2019 (COVID-19) pandemic, the use of full personal protective equipment (PPE) is advocated for patients undergoing emergency surgery in whom the infection status is unknown. This study aims to determine whether PPE has any influence on the rate of surgical site infection (SSI) in patients undergoing emergency exploratory laparotomy.

Methodology

Medical records of operated emergency cases in the general surgery department from 1st April 2020 to 24th February 2021 were studied. The surgeries done were divided into two groups: those done with full PPE (group A) and those done without full PPE (group B). The various parameters studied were the patient demography, presence of comorbidities, diagnosis, surgery done, class of surgery performed, the use of PPE, the post-operative presence, and type of SSI. Statistical analysis was done using SPSS software version 27.0 (IBM Corp., Armonk, NY). Chi-squared test was used to find the association of SSI with the use of PPE. Fisher’s exact test was used to explore the association between SSI with various comorbidities, surgery performed, and the class of surgery performed.

Results

A total of 126 patients underwent emergency exploratory laparotomy during the study period. A total of 61 patients were in group A and 65 patients in group B. A significant association was noted between the use of full PPE and the development of SSI (p = 0.032). Diabetes mellitus, history of alcohol intake, and the class of surgery performed were found to be significantly associated with the development of SSI.

Conclusion

A significant association in the occurrence of SSI with the use of full PPE was observed.

## Introduction

There have been reported cases of coronavirus disease 2019 (COVID-19) amounting to 170 million worldwide with 28 million occurring in India (as of June 2021). The American College of Surgeons has provided recommendations for the proper functioning of surgical care during the pandemic. Non-operative management is preferred for patients suspected of COVID-19, if feasible, for patient management. However, if operative management is planned, the use of appropriate personal protective equipment (PPE) must be utilized. Acute intestinal obstruction, bowel ischemia, hollow viscus perforation, and obstructed or strangulated hernia should proceed with emergency exploratory laparotomy with adequate precautions [1]. The Royal College of Surgeons, England recommends that all aerosol-generating procedures on suspected cases must be done using disposable fluid repellent PPE, gown, FFP3 respirators, eye and face protection, and disposable plastic apron [2].

Surgical site infection (SSI) is one of the most important causes of hospital-acquired infection. According to the CDC, SSI accounts for 46.4% of all hospital-acquired infections, thereby causing a significant toll on patient recovery and resources spent for the same [3]. The mortality rate associated with SSI is about 3%, whereas 75% of post-surgical deaths associated with SSI are directly attributable to SSI [4]. The WHO has recommended global guidelines for the prevention of SSI, which include pre-operative bathing, antibiotic prophylaxis, alcohol-chlorhexidine skin decontamination, skin barriers, and maintenance of intra-operative normothermia [5].

The effect of the use of PPE on the occurrence of SSI has not been studied so far. Here, we study the relation between the use of PPE and the occurrence of SSI in all operated cases of emergency exploratory laparotomy.

## Materials and methods

Study design and setting

It was a retrospective record-based study. The surgeries done were grouped into two groups. Group A included all cases done with full PPE and group B included all those done without full PPE.

Study population

All the cases of emergency laparotomies done in the department of general surgery at a tertiary care centre from 1st April 2020 to 24th February 2021 meeting the inclusion criteria were included in the study.

Inclusion and exclusion criteria

Inclusion criteria included patients of age 18 years and above and patients who underwent emergency exploratory laparotomy. Exclusion criteria included patients with incomplete medical records.

Collection of data and data analysis

The data were retrieved by electronic records and tabulated into Excel sheets (Microsoft Corporation, Redmond, WA) after taking clearance from the Institutional Ethics Committee, All India Institute of Medical Sciences, Rishikesh (AIIMS/IEC/21/279). The variables collected in the study included age, sex, presence of comorbidities (diabetes, hypertension, and hypothyroidism), addictions (smoking and alcoholism), diagnosis, surgeries done, class of surgery performed, use of PPE, post-operative presence of SSI, and the type of SSI. The tabulated data were analysed using SPSS software version 27.0 (IBM Corp., Armonk, NY).

The t-test was used to find the association of SSI with age. The chi-squared test was used to explore the association between "surgical site infection" with gender, diabetes, and the use of PPE. Fisher's exact test was used to explore the association between SSI with history of hypertension, hyperthyroidism, smoking, alcohol intake, diagnosis, surgeries performed, and the class of surgery performed. Regression analysis was carried out on the various factors with a significant association with SSI, and multivariate and univariate analysis was carried out for the same.

## Results

A total of 126 patients underwent emergency exploratory laparotomy in the Department of Surgery at All India Institute of Medical Sciences, Rishikesh from 1st April 2020 to 24th February 2021. Of the patients, 61 patients (48.4%) were operated with full PPE and 65 patients (51.6%) were operated without PPE. The demography of all patients included in the study, comorbidities, diagnosis, class of surgery, presence of SSI, and the type of SSI are summarized in Table 1.

**Table 1:** Summary of all relevant parameters

All parameters	Mean ± SD || Median (IQR) || Min-Max || Frequency (%)
Personal protective equipment (PPE)	
Used	61 (48.4%)
Not used	65 (51.6%)
Age (years)	43.75 + 14.50 || 42.50 (34.25-52.00) || 18.00-84.00
Gender	
Male	81 (64.3%)
Female	45 (35.7%)
Diabetes (present)	16 (12.7%)
Hypertension (present)	11 (8.7%)
Hypothyroidism (present)	4 (3.2%)
History of smoking (present)	14 (11.1%)
History of alcohol intake (present)	8 (6.3%)
Diagnosis	
Prepyloric perforation	35 (27.8%)
Acute intestinal obstruction	20 (15.9%)
Ileal perforation	19 (15.1%)
Duodenal perforation	7 (5.6%)
Small bowel gangrene	7 (5.6%)
Jejunal perforation	4 (3.2%)
Pyoperitoneum	4 (3.2%)
Appendicular perforation	3 (2.4%)
Gastric outlet obstruction	3 (2.4%)
Gastric perforation	2 (1.6%)
Others	22 (17.5%)
Class of surgery	
Clean	12 (9.5%)
Clean contaminated	15 (11.9%)
Contaminated	12 (9.5%)
Dirty	87 (69.0%)
Surgical site infection (SSI) (present)	42 (33.3%)
Type of SSI	
None	84 (66.7%)
Superficial SSI	15 (11.9%)
Deep SSI	10 (7.9%)
Organ space SSI	17 (13.5%)

There is a significant association between the use of PPE with the occurrence of SSI (χ2 = 4.592, p = 0.032; Table 2). However, there was no significant association between the use of PPE and the type of SSI (χ2 = 5.694, p = 0.127; Table 3). The organ space SSI showed a higher occurrence in surgeries done with full PPE compared to those done without the full PPE (19.7% vs. 7.7%; Table 3). An assessment of the association of SSI with various parameters was done. There was a significant association between SSI and diabetes mellitus (χ2 = 18.936, p < 0.001). Similarly, a significant association was found between SSI and history of alcohol intake (χ2 = 6.674, p = 0.016). A statistically significant association was also found between SSI and the class of surgery (χ2 = 8.868, p = 0.018). However, no significant association was found between SSI and age, gender, hypertension, smoking, or surgery performed (Table 4). Multivariate regression analysis was done for the dependent variable using all the predictor variables together. The odds ratio for the development of SSI with the use of PPE was 2.5, diabetes was 12.3, and with a history of alcohol intake was 4.9.

**Table 2:** The association between the use of personal protective equipment (PPE) with surgical site infection (SSI)

Surgical site infection	PPE			Chi-squared test	
	Used	Not used	Total	χ2	P-value
Present	26 (42.6%)	16 (24.6%)	42 (33.3%)	4.592	0.032
Absent	35 (57.4%)	49 (75.4%)	84 (66.7%)		
Total	61 (100.0%)	65 (100.0%)	126 (100.0%)		

**Table 3:** The association between the use of personal protective equipment (PPE) with the type of surgical site infection (SSI)

Type of surgical site infection	PPE			Chi-squared test	
	Used	Not used	Total	χ2	P-value
None	35 (57.4%)	49 (75.4%)	84 (66.7%)	5.694	0.127
Superficial SSI	9 (14.8%)	6 (9.2%)	15 (11.9%)		
Deep SSI	5 (8.2%)	5 (7.7%)	10 (7.9%)		
Organ Space SSI	12 (19.7%)	5 (7.7%)	17 (13.5%)		
Total	61 (100.0%)	65 (100.0%)	126 (100.0%)		

**Table 4:** The association between surgical site infection and various parameters *** Significant at p < 0.05; ^1^ t-test; ^2^ chi-squared test; ^3^ Fisher's exact test.

Parameters	Surgical site infection		P-value
	Present (n = 42)	Absent (n = 84)	
Age (years)	41.83 ± 14.24	44.71 ± 14.62	0.292^1^
Gender			1.000^2^
Male	27 (64.3%)	54 (64.3%)	
Female	15 (35.7%)	30 (35.7%)	
Diabetes (present)***	13 (31.0%)	3 (3.6%)	<0.001^2^
Hypertension (present)	4 (9.5%)	7 (8.3%)	1.000^3^
Hypothyroidism (present)	0 (0.0%)	4 (4.8%)	0.300^3^
History of smoking (present)	7 (16.7%)	7 (8.3%)	0.228^3^
History of alcohol intake (present)***	6 (14.3%)	2 (2.4%)	0.016^3^
Diagnosis			0.075^3^
Prepyloric perforation	9 (21.4%)	26 (31.0%)	
Acute intestinal obstruction	3 (7.1%)	17 (20.2%)	
Ileal perforation	10 (23.8%)	9 (10.7%)	
Duodenal perforation	2 (4.8%)	5 (6.0%)	
Small bowel gangrene	2 (4.8%)	5 (6.0%)	
Jejunal perforation	2 (4.8%)	2 (2.4%)	
Pyoperitoneum	3 (7.1%)	1 (1.2%)	
Appendicular perforation	1 (2.4%)	2 (2.4%)	
Gastric outlet obstruction	0 (0.0%)	3 (3.6%)	
Gastric perforation	2 (4.8%)	0 (0.0%)	
Others	8 (19.0%)	14 (16.7%)	
Class of surgery***			0.018^3^
Clean	0 (0.0%)	12 (14.3%)	
Clean contaminated	3 (7.1%)	12 (14.3%)	
Contaminated	5 (11.9%)	7 (8.3%)	
Dirty	34 (81.0%)	53 (63.1%)	

## Discussion

SSI has been extensively studied and various guidelines have been formulated over the years. In the era of the COVID-19 pandemic, the use of full PPE has been advocated. However, there are no studies currently done to study the impact of using full PPE in emergency laparotomies with SSI. The CDC defines SSI as the infection related to a surgical procedure that occurs within 30 days following surgery near the surgical site or 90 days following surgery where an implant is involved [6]. SSIs are classified into three groups, superficial, deep, and organ space/body cavity, with specific criteria to categorize SSIs into the respective class. Risk factors have also been extensively studied and can be broadly classified into patient-dependent and surgery-dependent factors. Patient-dependent factors include age, nutritional status, history of diabetes, smoking, obesity, concomitant infection, and immunocompetent status; whereas surgery-dependent factors include adequate hygiene and skin disinfection, hair shaving, perioperative antibiotics, operating room air conditioning, instrument sterilization, sterile gloving and gowning, type of surgery, haemostasis, and significant surgical trauma [7].

The full PPE kit available in our institution is non-sterile, as is the case in most developing countries. Following the application of this, the PPE is cleaned with a chlorhexidine-alcohol solution. A sterile gown is placed over the PPE as done in routine cases. Diagnosis of SSI was made according to the 2021 CDC guidelines [4]. Analysis of the data revealed a statistically significant association between the occurrence of SSI with the history of diabetes, alcohol intake, and the class of surgery done (more occurrence in dirty surgery, which accounted for most of our cases of about 69%). Similarly, on studying the impact of PPE on SSI, a statistical significance occurrence of SSI was noted in the surgeries done with the use of a full PPE (χ2 = 4.592, p = 0.032).

Mc Geehan et al. have mentioned a significant deficit in the world literature relating to emergency exploratory laparotomy and SSI in 2021 [8]. The high load of SSI in low-income countries compared to its counterparts in high and middle-income countries has been published by GlobalSurg Collaborative study in 2018. This study included 193 hospitals in 30 countries with 12,539 patients. The incidence of SSI varied between countries and type of surgeries performed with the highest incidence of SSI seen in the dirty class of gastrointestinal surgeries in the low-income countries at 39.8%, 31.4% in middle-income countries, and 17.8% in high-income countries [9]. Our study shows an SSI rate of 33% in emergency midline laparotomies. There is also an increased association of organ space SSI in cases done with full PPE (19.7%) compared to 7.7% done without a full PPE, though no statistical significance was identified (p = 0.127). However, an evaluation of the impact of PPE on SSI was not done in the GlobalSurg Collaborative study, mainly because this study was done much before the pandemic. Emphasis on the role of full PPE with N-95 masks came up during the COVID-19 pandemic, which had led to a paradigm shift in the way emergency surgeries were carried out.

After surgery, wound hygiene must be ensured with a "non-touch" technique. Wound dressing with sterile saline is advised and the use of antimicrobial products to reduce infection is not advised as proven in a randomised controlled trial done by Kamath et al., which shows no significant reduction in SSI with the use of chloramphenicol topical ointment [10]. Classical signs of SSI include redness, pain, raised temperature, oedema, and purulent discharge. The wound is opened, and pus is drained, with debridement advised if necrosis is detected in the wound. Relevant microbiological investigations including aerobic culture-sensitivity guided antibiotics are advised for the management of SSI. The role of the use of topical povidone-iodine in the prevention of SSI was a matter of debate for much of the past. A meta-analysis done by Lopez-Cano et al. in 2019 revealed no conclusive evidence of the occurrence of reduction of SSI with the use of topical povidone before the wound closure [11]. Nowadays, negative pressure wound therapy may be considered for the same.

## Conclusions

The present study shows a significant association in the occurrence of SSI with the use of full PPE. This might be attributed to the unsterile nature of the full PPE currently available in markets of developing nations. Hence, the authors recommend the use of sterile full PPE with the usual sterile surgical gown to reduce the occurrence of SSI. Further studies with a larger sample size are advocated for a more extensive search on this topic.
